# Initial experience and clinical comparison of two image guidance methods for SBRT treatment: 4DCT versus respiratory‐triggered imaging

**DOI:** 10.1120/jacmp.v12i3.3429

**Published:** 2011-01-31

**Authors:** Brian Wang, Prema Rassiah‐Szegedi, Hui Zhao. Jessica Huang, Vikren Sarkar, Martin Szegedi, Kristine E. Kokeny, Christopher J. Anker, Dennis C. Shrieve, Bill J. Salter

**Affiliations:** ^1^ Department of Radiation Oncology Huntsman Cancer Institute, University of Utah Salt Lake City UT 84112 USA

**Keywords:** image guidance, SBRT, 4DCT, respiratory‐triggered imaging

## Abstract

For Stereotactic Body Radiation Therapy (SBRT) treatment of lung and liver, we quantified the differences between two image guidance methods: 4DCT and ExacTrac respiratory‐triggered imaging. Five different patients with five liver lesions and one lung lesion for a total of 19 SBRT delivered fractions were studied. For the 4DCT method, a manual registration process was used between the 4DCT image sets from initial simulation and treatment day to determine the required daily image‐guided corrections. We also used the ExacTrac respiratory‐triggered imaging capability to verify the target positioning, and calculated the differences in image guidance shifts between these two methods. The mean (standard deviation) of the observed differences in image‐guided shifts between 4DCT and ExacTrac respiratory‐triggered image guidance was left/right (L/R)=0.4(2.0)mm, anterior/posterior (A/P)=1.4(1.7) mm, superior/inferior (S/I)=2.2(2.0) mm, with no difference larger than 5.0 mm in any given direction for any individual case. The largest error occurred in the S/I direction, with a mean of 2.2 mm for the six lesions. This seems reasonable, because respiratory motion and the resulting imaging uncertainties are most pronounced in this S/I direction. Image guidance shifts derived from ExacTrac triggered imaging at two extreme breathing phases (i.e., full exhale vs. full inhale), agreed well (less than 2.0 mm) with each other. In summary, two very promising image guidance methods of 4DCT and ExacTrac respiratory‐triggered imaging were presented and the image guidance shifts were comparable for the patients evaluated in this study.

PACS number: 87.55.ne

## I. INTRODUCTION

Hypofractioned stereotactic body radiation therapy (SBRT) is being increasingly utilized in lung and liver tumor treatment and several recent studies have reported promising results.^(^ ^1^ ^–^ ^8^ ^)^ Image guidance is a key component in treatment setup because of the high conformal dose and typically small margins.^(9)^ Cone‐beam computed tomography (CBCT) is one of the most popular image guidance methods, and the target on CBCT is usually compared with the internal target volume (ITV) generated from the maximum intensity projection (MIP) of the simulation four‐dimensional (4D) scan to determine the image guidance shifts.^(^ ^10^ ^–^ ^15^ ^)^ Due to the nature of slow blurred scan, CBCT is not the ideal way to visualize tumor to determine image guidance shifts. In order to address this issue, some researchers are working on respiratory‐correlated 4D CBCT for better appreciation of the tumor motion at the time of treatment setup, and thus producing more comparable image series with the original simulation 4DCT scan. However, such technologies have not yet been made commercially available.^(^ ^16^ ^,^ ^17^ ^)^

At our institution, in addition to acquiring an initial simulation 4DCT used for definition of the ITV, we also routinely acquire a ‘control’ image guidance 4DCT scan in the CT suite immediately prior to each treatment fraction for comparison with the simulation‐derived ITV. This comparison is used to derive the required image guidance shifts. When using our Novalis treatment unit for treatment delivery, we also have the ability to acquire ExacTrac (BrainLAB AG, Feldkirchen, Germany) stereoscopic planar images in “respiratory‐triggered” mode to derive similar image guidance shifts for patients with implanted fiducial markers. The ExacTrac respiratory‐triggered mode has been previously presented in the literature for SBRT treatment of liver and lung tumors.^(^ ^18^ ^,^ ^19^ ^)^ Here we report our experience in using these two methods (i.e., 4DCT and ExacTrac respiratory‐triggered imaging), and we quantify the differences in image‐guided shifts between these two methods. We also report the difference between full exhale and full inhale derived image guidance for ExacTrac respiratory‐triggered images, and present our findings on pre‐ and post‐treatment (i.e., intrafractional) shifts when using ExacTrac triggered imaging.

### II. Materials and Methods

### A. 4DCT image guidance for SBRT

On the day of simulation, patient is immobilized inside the whole‐body BodyFIX system (Medical Intelligence Medizintechnik GmbH, Schwabmünchen, Germany) with an abdominal compression pillow and double vacuum seal. Two side ball bearings (BBs) are placed on the BodyFIX bag and one BB is placed on the patient's skin near the xiphoid process in alignment with CT suite room lasers. Triangulation of these three BBs gives the setup isocenter. Two lines are drawn on the superior and inferior ends of the bag in alignment with the sagittal room laser. The CT table is lowered to an appropriate position for acquiring the scan and the scanning height is recorded. All the patients are scanned on a LightSpeed RT CT scanners (GE Health Care, Waukesha, WI) using our department 4DCT scan protocols, at 0.5 sec per revolution gantry rotation speed, and 1.25 mm slice thickness, 0.5 mm×0.5 mm axial pixel size at 120 kV. The real‐time position management (RPM) block (Varian Oncology Systems, Palo Alto, CA) is placed on the patient's chest wall to produce the respiratory input signal for the purposes of binning the images.^(20)^ The 4DCT raw data is processed using the GE 4D software into 10 separate phase‐binned images^(21)^ and the ITV is contoured directly using the 10 phases from the simulation 4DCT dataset.

**Figure 1:**
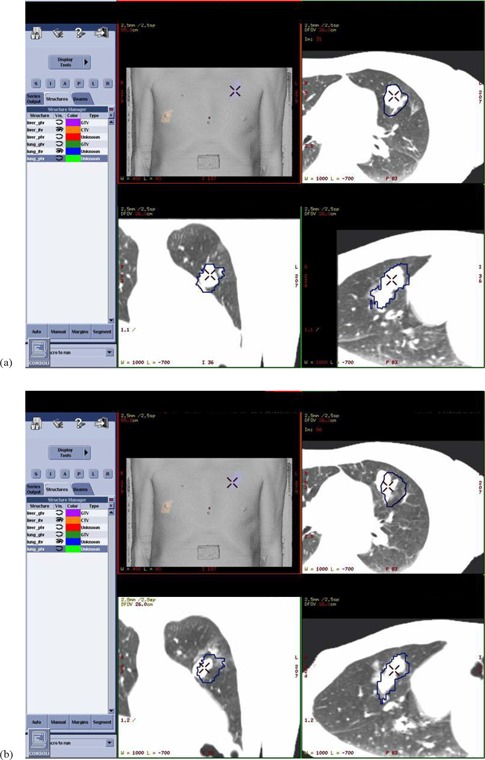
4DCT images: (a) at full exhale respiratory phase on simulation day, and (b) at full exhale respiratory phase on treatment day #4 for one patient (case #6) in this study. The contoured ITV contained the tumor motion envelope on the day of simulation, but the tumor can be seen to have shifted for treatment day #4. Image guidance shifts were carried out to align to the target's day #4 location prior to treatment delivery. In this example, the corrective shifts were 2 mm posteriorly and 9 mm to patient left.

On the day of treatment, the immobilization bag is re‐aligned to the room lasers for the two BBs on the side of the bag and two sagittal lines, and then the CT table is lowered to the same scanning height as the simulation. This control 4DCT scan is registered to the simulation 4DCT dataset via a manual, or ‘physical’, registration method. This ‘physical’ registration is similar to the way that a PET and CT dataset are registered to each other, by virtue of having been scanned in the same position in the bore with, therefore, the same coordinate system origin. In our workflow, we ensure that both scans are acquired with identical coordinate system origins in the CT bore by careful alignment of the BodyFIX immobilization bag to CT suite room lasers each day. Following the control CT scan, the ITV and the simulation 4DCT image set are first loaded into the GE Advantage Workstation (AW version 4.4) ‐ SimMD software, and then the control 4DCT image set is subsequently loaded. We ensure that the physical registration of the two 4DCT datasets is accurate by confirming that the BBs placed at the bag/patient‐posterior interface are coincident in both 4DCT image sets. Having thus physically registered the BodyFIX bag to itself, any difference in tumor position is attributable either to mispositioning of the patient in the bag, or to movement of the tumor relative to bony anatomy within the patient. By measuring the deviation in tumor position on the dynamic 4DCT scans, an accurate determination of the required image‐guided shifts can be ascertained. Because the two image sets (simulation and control) share the same scanning coordinate system, the simulation‐defined ITV is also visible on the GE SimMD display of the control 4DCT dataset, thus facilitating evaluation of treatment day tumor position relative to the treatment‐planned ITV motion envelope. Figure 1 depicts 4DCT images at full exhale respiratory phase (a) on simulation day and (b) on one treatment day (#4) for one patient (case #6) in this study. The contoured ITV contained the tumor motion envelope on the day of simulation, but the tumor can be seen to have shifted for treatment day #4. Image guidance shifts were carried out to align to the target's day #4 location prior to treatment delivery. In this example, the corrective shifts were 2 mm posteriorly and 9 mm to patient left.

After the 4DCT acquisition, and subsequent derivation of the image guidance shifts, the patient is carefully transported by gurney from the CT suite to the treatment suite. To minimize the likelihood of patient movement during this transport, we transfer the patient from the CT suite to the treatment room within the BodyFIX device – employing the double vacuum component of the BodyFIX system utilized when transferring the patient between CT/treatment couch and gurney, to ensure that no patient movement occurs during the process. Additionally, a quality assurance (QA) check is performed in the treatment suite by using room lasers to ensure that a mark placed at the patient's sternum is still aligned to the isocenter mark on the BodyFIX system in the S/I and L/R directions, thus making it highly unlikely that patient movement during transport could go undetected. The transport process from the CT suite to the treatment suite covers approximately 200 feet and typically takes less than 1 minute.

### B. ExacTrac respiratory‐triggered imaging guidance

Because of the difficulty in visualizing most liver tumors on non‐contrast imaging, SBRT liver patients treated in our facility are routinely implanted with fiducial markers at the tumor periphery by our interventional radiologists to facilitate target identification on image guidance datasets. The implanted fiducial markers are 1×3 mm ACCULOC carbon soft tissue markers (CIVCO, Kalona, IA) to minimize image artifact on CT images. For these patients we also utilize ExacTrac respiratory‐triggered images to verify the in‐room target positioning by triggering image acquisition at a predetermined respiratory phase and comparing it to the digital reconstructed radiograph (DRR) generated at the same respiratory phase from the treatment planning 4DCT scan.

**Figure 2:**
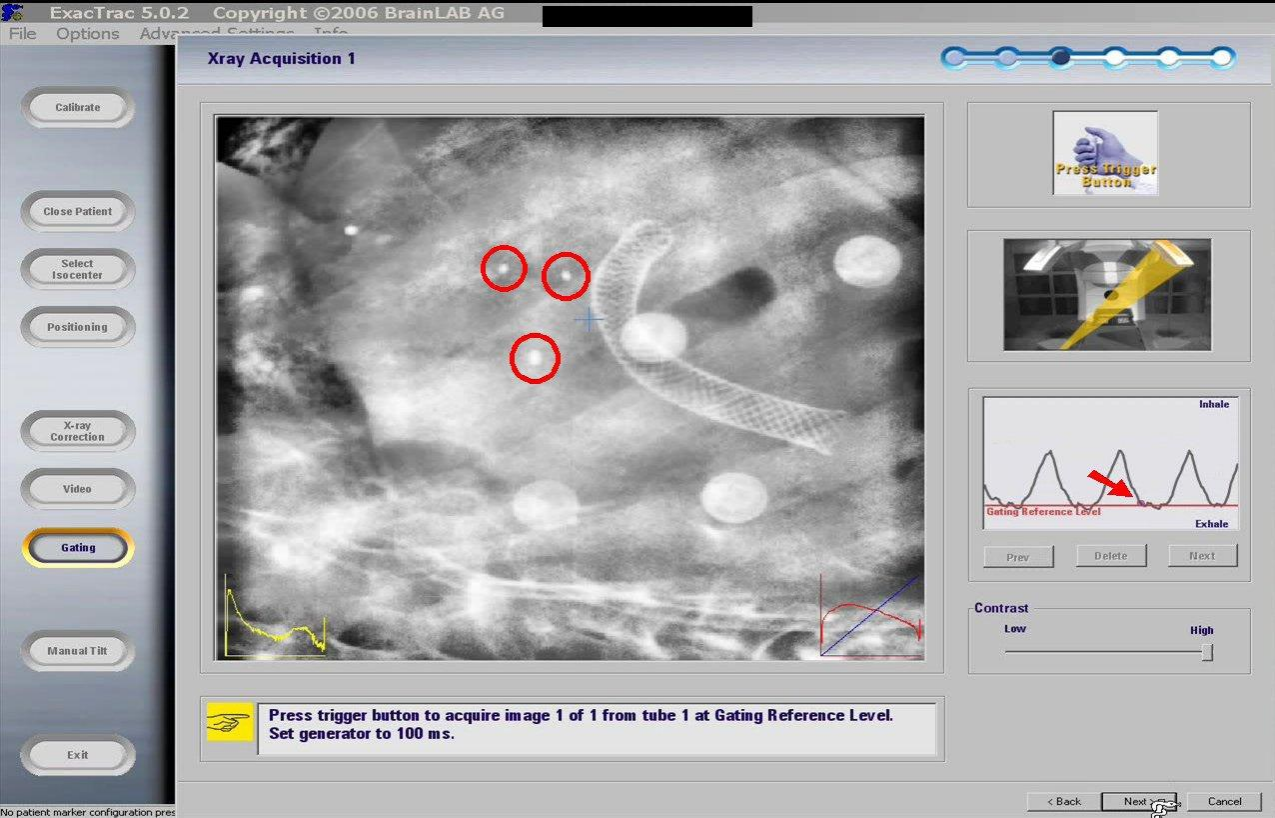
One of two ExacTrac images taken at the full exhale reference level (arrow) with three fiducial markers (indicated by circles).

**Figure 3:**
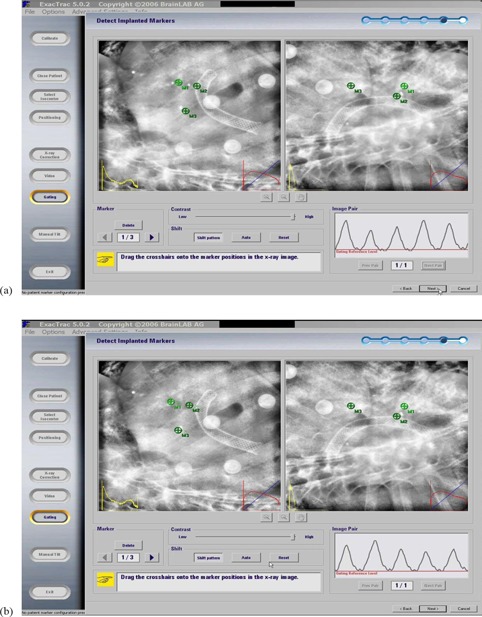
Two orthogonal X‐ray images acquired at the full exhale reference level of a liver tumor patient with the implanted fiducial markers before (a) and after (b) fusion. Markers (M1, M2, and M3) were reconstructed from planning CT of the location of the implanted fiducial markers and then were projected onto the X‐ray images.

Our work flow is as follows: The full exhale phase image set is transferred to the BrainScan (BrainLAB, Feldkirchen, Germany) treatment planning software and the target isocenter is then placed. The patient file is then exported to the ExacTrac software, and the implanted fiducial markers are identified in the software. A gating “reference level” corresponding to full exhale phase is selected in the ExacTrac software. Because the 4DCT image guidance process described early is our current clinical standard of practice, we always perform image‐guided correction based on the 4DCT shifts. After performing the 4DCT‐derived image guidance shifts in the treatment suite and immediately prior to treatment, five or six infrared body markers are placed on the patient's chest to generate a surrogate signal for the breathing pattern when tracked by two infrared cameras of the ExacTrac system. Two orthogonal kilovoltage X‐ray planar images are then triggered and acquired at the reference breathing level (Fig. 2). The expected locations and pattern of the implanted fiducial markers, reconstructed from the planning CT, are then projected onto the X‐ray images for manual identification/adjustment to facilitate calculation of required shifts by the ExacTrac software (Fig. 3). The identification of fiducial locations prior to fusion was done by a single physicist for consistency. It is noted that while we use the ExacTrac system to perform respiratory‐triggered image guidance, we do not currently perform gated treatment delivery because of our preference to treat using a conformal arc delivery method.

### C. Clinical workflow and comparison between the two image guidance methods

In our clinical workflow, patients are aligned using the lasers in the treatment room before treatment. The image guidance shifts obtained using the 4DCT method are then applied. After that, a pair of respiratory‐triggered ExacTrac X‐ray images is obtained and the internal fiducial markers are used to calculate the shifts, which are referred to as the image guidance shifts by the ExacTrac system. Because the image guidance shifts based on our 4DCT method have already been applied at this point in the process, the shifts calculated by the ExacTrac represent the difference between the two methods. It is noted that the ExacTrac shifts were not applied for patient treatment and thus the final treatment was based on the 4DCT image guidance shifts that were approved by physicians. However, good agreement between the two sets of image guidance shifts provides us with further clinical reassurance of a good treatment alignment.

### D. Patient selection and data acquisition

Patients with either liver or lung lesions treated by SBRT in our clinic, who also had internal implanted fiducial markers, were selected for this study. We report our experience in using the two image guidance methods described above for five different patients, with five liver lesions and one lung lesion for a total of 19 delivered SBRT fractions. All these ExacTrac respiratory‐triggered image guidance were acquired at the full exhale breathing phase. Additional ExacTrac images were acquired at the full inhale breathing phase for a subset of six fractions, and image guidance shifts were compared between the full inhale and the full exhale phases. To acquire the ExacTrac images at the full inhale breathing phase, a separate patient folder was first created and 4DCT image set at full inhale phase was transferred into this folder to generate the DRRs. The reference level for image acquisition at the ExacTrac workstation was also set to the full inhale phase.

### E. End‐to‐end phantom study

In order to quantify the baseline of the system localization accuracy, an end‐to‐end study was performed using the ET gating phantom (BrainLAB AG, Feldkirchen, Germany). This phantom includes two sections: main body and a simulated chest wall. The main body consists of 15 layers of 1 cm thick, 15 cm×15 cm acrylic slabs with several fiducial markers embedded. Both the main body and the simulated chest wall can be programmed to move independently from each other, driven by either sinusoidal or patient‐specific breathing traces. The same image guidance workflow for SBRT patients, as described previously, was repeated on the phantom moving with a 5‐sec cycle, 3 cm sinusoidal motion in S/I direction. Infrared markers were placed on the simulated chest wall as surrogates to track breathing motion at both 4DCT acquisition and ExacTrac triggered imaging system. Three experiments were carried out with the phantom: 1) at treatment isocenter location at full exhale phase; 2) at treatment isocenter location at full inhale phase; 3) with known shifts from isocenter at full inhale phase.

## III. RESULTS & DISCUSSION

### A. End‐to‐end phantom study

The three phantom experiments had less than a 1.0 mm difference in all three principle directions between the two image guidance methods: 4DCT vs. ExacTrac respiratory‐triggered imaging. The 3D vectors of difference were 1.1 mm, 0.8 mm and 1.4 mm for the full exhale, full inhale and known shifts experiments, respectively. Other groups have performed similar phantom studies to quantify the localization accuracy for the ExacTrac image guidance system. Willoughby et al.^(18)^ reported 1.7 mm localization accuracy on phantom for ExacTrac using a 20% phase gating window, and 1.4 mm localization accuracy on a static phantom. Yan et al.^(22)^ reported an average positioning accuracy of 1.0 mm using the Novalis Body system, but the gating feature was not available at the time of the study. Wurm et al.^(19)^ performed gated Winston‐Lutz tests on phantom and reported an overall system accuracy of 1.0 mm using 10% gating window for the ExacTrac gating system. In summary, our phantom studies agreed well with the existing body of literature, and we can establish the baseline accuracy for our study on a moving phantom is 1.0 mm in any principle direction, and less than 2.0 mm for the 3D vector. It is noted that our phantom study did not include gated radiation delivery component because we only use the ExacTrac respiratory‐triggered imaging feature for image guidance purposes in our clinical workflow.

### B. Image guidance shifts between 4DCT and ExacTrac triggered imaging

**Figure 4:**
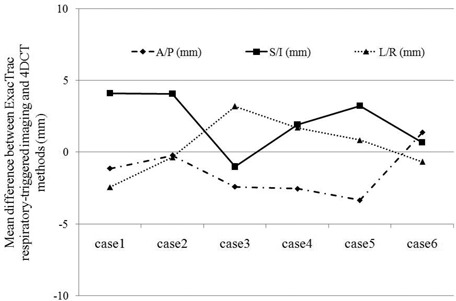
Mean differences between two image guidance methods: 4DCT and ExacTrac respiratory‐triggered images.

As shown in Table 1 and Fig. 4, the mean (standard deviation) of the observed differences in image‐guided shifts between 4DCT image guidance and ExacTrac respiratory‐triggered image guidance for all five patients, 6 lesions and 19 delivered fractions was L/R=0.4(2.0) mm, A/P=−1.4(1.7) mm,S/I=2.2(2.0) mm, with no difference larger than 4.1 mm noted in any given direction, for any individual case. The largest error (4.1 mm) occurred in the S/I direction and this is not unexpected, as respiratory motion and the resulting imaging uncertainties are most pronounced in this S/I direction. It is noted that the mean difference of 0.4 mm in the lateral direction was less than the system localization accuracy of 1.0 mm based on our phantom study and literature. This fact suggested that patient positions in the BodyFIX were maintained well during the transport from CT suite to treatment suite, since it was unlikely for patients to move only in S/I and A/P directions while holding still in L/R direction. And furthermore, as mentioned in Section II A above, all patients are confirmed by QA check of alignment to BodyFIX isocenter in the treatment suite, to have maintained their position relative to that ‘marked’ in the CT suite.

**Table 1:** Mean differences between two image guidance methods: 4DCT and ExacTrac respiratory‐triggered images.

*Case#*	*Site*	*A/P (mm)* ^a^,^b^	*S/I (mm)*	*L/R (mm)*	*Composite 3D (mm)*
1	liver	−1.1	4.1	−2.4	5.9
2	liver	−0.2	4.0	−0.4	4.2
3	liver	−2.4	−1.0	3.2	4.7
4	liver	−2.6	1.9	1.7	3.6
5	liver	−3.4	3.2	0.8	4.9
6	lung	1.4	0.7	−0.7	2.5
Mean±Standard Deviation		−1.4±1.7	2.2±2.0	0.4±2.0	4.3±1.2

a A/P= anterior/posterior; S/I= inferior/superior; L/R= left/right.

b The directions of ExacTrac image guidance shifts relative to 4DCT shifts were: +posterior; +superior; +right.

There are several possible explanations for the relatively minor disagreements between the two image guidance methods and these represent sources of further work. Possible contributing factors to error in the 4DCT images are: (a) 4DCT phase binning errors, (b) different patient breathing patterns at simulation vs. treatment day, and (c) image artifacts due to the velocity of the tumor. Potential factors contributing to ExacTrac respiratory‐triggered image errors are: (a) full exhale phase on ExacTrac software may not match full exhale phase amplitude of 4DCT dataset used to create the DRR for comparison (as indicated by the arrow in Fig. 2, it is possible for the patient full exhale breathing peak to surpass the image triggering level significantly, thus resulting in image aliasing due to higher tumor velocity than when the full exhale DRR was created); (b) the ExacTrac image acquisition time of 125 ms, coupled with a maximum 800 mA for the ExacTrac X‐ray tube, may not allow for sufficient mAs (100 mAs maximum) generation to, subsequently, allow for clear visualization of the fiducials for large patients (this could result in uncertainty in user interpretation of the fiducial locations); and (c) user error in manually defining the fiducial locations (as seen in Fig. 3(b), fiducial M3 did not match perfectly on both images and a compromise of the fusion had to be made considering all three fiducial markers). Another possible source for the differences in the two methods could be attributed to the fact that the patient position changes during transport from the CT suite, where the 4D image guidance CT scan was obtained prior to treatment, to the treatment suite. We implemented several measures as discussed in the Method Section above to minimize the likelihood of this occurrence. Because of this careful transfer and QA process, we believe it reasonable to interpret the differences between the 4D image‐guided shifts and the respiratory‐triggered image guidance process as being due to inherent differences in the data collected by the two methods.

### C. Full inhale versus full exhale shifts

For a subset of six fractions from three patients, ExacTrac respiratory‐triggered images were also acquired on the full inhale phase immediately after the ExacTrac images at the full exhale phase. These kilovoltage X‐ray images were then fused to the DRRs that were generated from the same full inhale phase on the simulation 4D CT scans to derive the image guidance shifts at the full inhale phase. Table 2 shows the differences between ExacTrac respiratory‐triggered image guidance shifts acquired from full exhale vs. full inhale phases for six different fractions in three patients. The mean of the differences in any of the three principle directions was less than 1.0 mm, with the maximum differences all being within 2.0 mm.

**Table 2:** Differences between two breathing phases: full exhale and full inhale, determined by ExacTrac respiratory‐triggered images.

*Case# : Fraction#*	*A/P (mm)* ^a^,^b^	*S/I (mm)*	*L/R (mm)*
case1 : fx3	−1.4	−0.8	−0.8
case3 : fx1	0.5	0.1	−0.6
case3 : fx2	0.7	−1.7	0.0
case3 : fx3	0.8	0.7	2.0
case3 : fx4	1.0	−1.4	0.8
case4 : fx1	0.4	−0.8	1.2
Mean	0.3	−0.6	0.4

a A/P=anterior/posterior; S/I=inferior/superior; L/R=left/right.

b The directions of ExacTrac image guidance shifts at full exhale phase relative to full inhale phase shifts were: +posterior; +superior; +right.

### D. Pre‐ versus post‐treatment image guidance shifts

For a subset of four fractions from four different patients, ExacTrac respiratory‐triggered images were also acquired after the treatment delivery at the same full exhale phase. The image guidance shifts derived from these ExacTrac images represented the post‐treatment image guidance shifts, and they were compared with those shifts from pre‐treatment ExacTrac images. Table 3 presents data derived for four treatment fractions where post treatment images were acquired to verify that no patient motion had occurred during treatment. All these pre‐ and post‐treatment derived image shifts were based on the full exhale phase. The mean of the differences of pre‐ and post‐treatment positions in any of the three principle directions were seen to be less than 1.0 mm, with maximum differences all less than 1.5 mm. This is consistent in magnitude with our observation of post‐treatment patient motion via laser to skin mark comparison.

**Table 3:** Differences between pre‐ and post‐treatment, determined by ExacTrac respiratory‐triggered images at full exhale phase.

*Case# : Fraction#*	*A/P (mm)* ^a^,^b^	*S/I (mm)*	*L/R (mm)*
case1 : fx2	−1.3	−0.5	0.2
case3 : fx2	−0.3	1.3	0.1
case4 : fx1	−1.4	0.1	−1.0
case6 : fx5	−0.1	−0.4	0.0
Mean	−0.8	0.1	−0.2

a A/P=anterior/posterior; S/I=inferior/superior; L/R=left/right.

b The directions of ExacTrac image guidance shifts at pre‐treatment relative to post‐treatment shifts were: +posterior; +superior; +right.

### IV. Conclusions

Two promising methods of image guidance for SBRT of liver and lung lesions were presented and compared. One is a novel method developed in our clinic that uses daily 4DCT imaging in the CT simulation suite to thoroughly characterize lesion motion prior to each delivered SBRT fraction. The second method uses ExacTrac respiratory‐triggered imaging capability to confirm the position of the targeted lesion on the treatment table immediately prior to treatment, for lesions where internal fiducial markers have been implanted. We compared the two methods and found them to agree within reasonably expected limits. Potential contributing factors to the disagreement were presented and will be studied in future work. The ExacTrac images triggered at two extreme breathing phase, (i.e., full exhale vs. full inhale) were seen to agree within 2.0 mm. This work should serve as valuable confirmation of the accuracy of each of these two promising methods for users considering use of either, or both, methods.
